# Connected Research: The Potential of the PID Graph

**DOI:** 10.1016/j.patter.2020.100180

**Published:** 2021-01-08

**Authors:** Helena Cousijn, Ricarda Braukmann, Martin Fenner, Christine Ferguson, René van Horik, Rachael Lammey, Alice Meadows, Simon Lambert

**Affiliations:** 1 DataCite, Welfengarten 1B, 30167 Hannover, Germany; 2 Data Archiving and Networked Services, Anna van Saksenlaan 51, 2593 HW Den Haag, the Netherlands; 3 The European Bioinformatics Institute, Wellcome Genome Campus, Hinxton, Cambridgeshire CB10 1SD, UK; 4 Crossref, New Road, Oxford OX1 1BY, UK; 5 National Information Standards Organization (NISO), 3600 Clipper Mill Road Suite 302, Baltimore, MD 21211-1948, USA; 6 UKRI-STFC, Scientific Computing Department, Rutherford Appleton Laboratory, Harwell Campus, Didcot OX11 0QX, UK

**Keywords:** DSML 4: Production: Data science output is validated, understood, and regularly used for multiple domains/platforms

## Abstract

Persistent identifiers (PIDs) provide unique and long-lasting references to entities. They enable unique identification persistently over time and hence play a crucial role in supporting the FAIR (Findable, Accessible, Interoperable, Reusable) principles. In this paper, we describe how the benefits of PIDs can be amplified by connecting them via their metadata. We are introducing the next step in PID infrastructure: the PID Graph. The PID Graph establishes connections between different entities within the research landscape, thereby enabling both researchers and institutions to access new information. The paper closes with three recommendations, which will help to optimize the use and value of PIDs within the research ecosystem.

## The Bigger Picture

PIDs provide unique and long-lasting references to entities and play a crucial role in research infrastructure. They enable unique identification persistently over time and contribute to making research entities more FAIR (Findable, Accessible, Interoperable, and Reusable).

The benefits of PIDs can be amplified by connecting them via their metadata. Therefore, we are introducing the next step in PID infrastructure: the PID Graph. The PID Graph establishes connections between different entities within the research landscape, thereby enabling researchers and institutions to access new information.

Through the PID Graph, the infrastructure is in place to answer new questions about connections within the research world. However, these will only have meaningful answers if sufficient information is present within the PID Graph. Therefore, the paper closes with three recommendations for different stakeholders, which will help to optimize the use and value of PIDs within the research ecosystem.

## Teaser

Persistent identifiers (PIDs) provide unique and long-lasting references to entities. They enable unique identification persistently over time and play a crucial role in supporting the FAIR principles. In this paper, we describe how the benefits of PIDs can be amplified by connecting them via their metadata. We are introducing the next step in PID infrastructure: the PID Graph. The PID Graph establishes connections between different entities within the research landscape, thereby enabling both researchers and institutions to access new information.

## Introduction

The need for sharing research findings and integrating existing information to facilitate new discoveries is more evident than ever. With major steps being taken toward the implementation of transcontinental infrastructures such as the European Open Science Cloud (EOSC), AmeliCA, and several national infrastructures to share research outputs, it is timely to examine the use of identifiers and metadata in connecting research. Taking the EOSC as an example, Persistent identifiers (PIDs) are a prominent component of the European scientific ecosystem,^1^ and a PID policy for the EOSC has been formulated.^2^

To enhance the value of research, resources should be made FAIR (Findable, Accessible, Interoperable, and Reusable).^3^ To enable a FAIR research landscape, a technical infrastructure is needed that allows digital information to be found and accessed in a reliable and sustainable manner.^4^ PIDs are a crucial aspect of this technical infrastructure, playing a role in each of the FAIR elements.^5^

A PID is a unique and long-lasting reference to an entity, such as a dataset, paper, or person. It is a machine-readable string of characters, which conforms to a defined lexical scheme and must be associated with one, and only one, entity within the world.^6^ Unlike a uniform resource locator (URL), a PID reliably points to that specific entity over time, thus ensuring that it can always be found. Should an entity disappear, the PID will still point to essential information about it, although it may now be represented by a tombstone page, a special type of landing page stating that an entity has been removed. In the FAIR principles,^3^^,^^5^ PIDs are explicitly mentioned in several of the FAIR facets. The first and third facet of the Findable principle, F1 (“(Meta)data are assigned a globally unique and persistent identifier (PID)”) and F3 (“Metadata clearly and explicitly include the identifier of the data it describes”), highlight the importance of PIDs for making resources findable. PIDs are also mentioned in the Accessibility facet A1 (“(Meta)data are retrievable by their identifier using a standardized communications protocol”). Furthermore, PIDs improve interoperability by creating links between digital entities and providing context through metadata with references to other metadata. Finally, PIDs play a role in the reusability of data by enabling rich metadata and provenance to be associated with a digital object.^7^ PIDs are also a crucial component in the FAIR Digital Object model. A FAIR Digital Object (FDO) is a self-contained, machine-actionable data package that binds all critical information about an entity in one place. Importantly, FDOs are accessed through their PID.^8^ The presence of PIDs thus plays a crucial role in all aspects of FAIR and FDOs, as well as in evaluating the FAIRness of resources.^9^

To function properly, PIDs need to be supported by an infrastructure that provides some guarantee that the form and properties of the PID can be maintained over time. This requires a long-term commitment to maintain the service by an organization that is equipped to make such a commitment. Additionally, a unique name alone does not make a PID valuable. Rather, the associated metadata describing the entity, agreed standards, associated services, and the community around PIDs are crucial to ensuring that an identifier can be used and adds value to the digital infrastructure. This paper describes the role of PIDs in realizing open research, as well as the efforts made by PID service providers to sustain the PID infrastructure. Most importantly, we introduce the PID graph and outline how connecting PIDs can provide opportunities to link entities and answer a variety of research questions and use cases. We provide three recommendations to optimize the use and value of PIDs in the scientific infrastructure.

## PID Services

### Definition of PID Services

PIDs can be assigned to a wide range of scholarly entities such as publications, datasets, organizations, and researchers. We define a PID service as a service that registers PIDs and their associated entities. In addition to the characteristics mentioned above, schemes for digital PIDs will typically have additional properties:

• They are resolvable via HTTPS uniform resource identifiers (URIs) to allow the discovery of the entity, or a digital proxy. This may include content negotiation to find different manifestations of the entity.
• They contain searchable metadata that describe some properties of the entity to aid the effective use of that PID.

The overall PID infrastructure is made up of PID service providers, repositories, curation systems, aggregators, indexes, metadata, and standards—and people. PIDs connect all of these elements, not only technically, via metadata and integrations, but also socially, via communities that have formed over decades or longer.^10^ Depending on the policy and governance principles, multiple options are available for entities such as datasets, publications, researchers, organizations, and software. Information sources that can help select a PID are the Dutch Pidwijzer (“PID Pointer” [see https://www.pidwijzer.nl/en]) and the PID guides created by the FREYA project.^11^ It is important to keep in mind that a PID is only as good as the services built around it, and the PID services are only as good as the social adoption and sustainability they can achieve.

### PIDs for Different Entities

The foundational PID infrastructure mentioned above provides a layer upon which to build new PIDs for new entities that people want to be able to uniquely identify and use, and may want to use well into the future.

This spectrum is captured by a landscaping report^12^ to understand the range of research entities that can be identified and referenced with PIDs. A maturity matrix (Table 1) was devised to provide a sense of how advanced the identifier infrastructure is around a particular entity. The maturity scale identified three steps: mature, emerging, and immature identifier infrastructure.

**Table 1:** A Landscape Analysis of Research Entities, Identifying PID Types and Infrastructure Maturity (as of May 2018)

Research Entity	PID Types Used	Maturity of PID Infrastructure
Publication	DOI, accession number, handle, URN, Scopus EID, Web of Science UID, PMID, PMC, arXiv identifier, BibCode, ISSN, ISBN, PURL	mature
Citation	OCI (secondary aggregation of information)	emerging
Conference	DOI, accession number	emerging
Researcher (or scholar)	ORCID IDs, ISNI (also DAIs, VIAFs, arXivIDs, OpenIDs, ResearcherIDs, ScopusIDs)	mature
Organization	DOI, ISNI, GRID, Ringgold IDs, ROR IDs	emerging
Data	DOI, accession number, handle, PURL, URN, ARK	mature
Data repository	none	immature
Grants	DOI, PURL	emerging
Project	local identifier, accession number, RaiD	emerging
Experiment	none	immature
Investigation	DOI, accession number	emerging
Analysis	GitHub gist	immature
Software	DOI, SHA-1 hash	emerging
Computer simulation	UUID	emerging
Software license	none	immature
Equipment		
Instrument, device, sensor, platform, research facility	DOI, RRID, UID	emerging
Archival/storage facility	URI, DOI, UUID	emerging
Field station	none	immature
Sample		
Geological or biological sample	accession number, RRID, DOI, IGSN	emerging
Cultural artifact	DOI, URN, accession number	emerging
Historical or mythical person	URI	emerging
Temporal period and historical place	ARK, URI, accession number	immature
Study Registration		
Clinical trial; non-clinical registration	accession number, DOI	immature
Data management plan	DOI	immature
Workflow	URI, DOI	immature
Protocol	DOI	immature

Adapted from Ferguson et al.[12]. ARK: Archival Resource Key, BibCode: Bibliographic Codes, DAI: Digital Author Identifier, DOI: Digital Object Identifier, ID: Identifier, IGSN: International Geo Sample Number, ISBN: International Standard Book Number, ISNI: International Standard Name Identifiers, ISSN: International Standard Serial Number, OCI: Open Citation Identifier, ORCID: Open Researcher and Contributor ID, PMID: PubMed ID, PURL: Persistent Uniform Resource Locators, RAiD: Research Activity identifier, RRID: Research Resource ID, SHA-1: Secure Hash Algorithm 1, UUID: Universally Unique Identifiers, URI: Uniform Resource Identifier, URN: Uniform Resource Name, VIAF: Virtual International Authority Files

In Table 1, mature indicates that the infrastructure is in common research community use, i.e., regular use within a research discipline. An emerging infrastructure is not yet available for use by the common research community; services may be in pilot or being actively planned by a working group. For an immature infrastructure, there are only nascent discussions; there is no definite community consensus on how to proceed with implementing PIDs for this research resource.

The PID infrastructures (and their ensuing community adoption) require time to develop: a service simply to register a PID alone is not enough—the PID needs to have a flexible metadata schema that can evolve. It also needs to be machine-readable by multiple systems and be integrated into stakeholder workflows, ultimately mapping and interoperating with all other relevant PIDs and PID services. The overview of PID types and PID infrastructures in Table 1 is not exhaustive. As can be seen from the table, many research resources still require PIDs as well as PID services.

### The Role of Communities and Adoption of PID Services

Adoption of PID services is a measure of community endorsement and, in a “needs-driven” approach to PID service development, it is important to seek community advice and ongoing appraisal of development early in the process. Several PID service providers, such as DataCite, ORCID, and Crossref, are member organizations, and an active engagement with and involvement of the member communities plays an important role in the adoption of PID services. In addition, PID-specific forums for engaging with community stakeholders have successfully been established. PIDapalooza (https://pidapalooza.org/), a conference on PIDs organized by several PID service providers, has been held annually since late 2015. The European project FREYA (https://www.project-freya.eu/en), in which several institutions working with PIDs collaborate, established pidforum.org in 2019. This is an online global information and discussion platform for all matters relating to PIDs, which is used to engage with the wider community. Lastly, the Research Data Alliance (RDA; https://rd-alliance.org/) has several working groups and interest groups concerning PIDs that—together with the RDA plenary meetings—allow members across disciplines, who have an interest in PID services relating to their community, to come together. These meetings and discussion groups are effective in bringing together seemingly diverse disciplinary groups around unified themes.^13^

## The Value of PIDs

### How PIDs and Metadata Support Research

PIDs and their associated metadata are critical enablers for research and scholarship, and have capabilities that support the aims of open research, hence their recognition in the FAIR principles.^3^ To include and expand on the FAIR principles, PIDs and metadata help ensure that the entities they refer to are:

• Discoverable and accessible: uniquely identifying and resolving objects, people, research institutions, and funding reliably and consistently, and linking them together. This makes it easier to find the correct entity and verify that it has been found, building trust in the research record.
• Usable and citable: pointing directly to an object, such as a specific item or a specific version of a dataset, increases the usability of that object for researchers. It also helps them formally cite research outputs such as data and resources (as opposed to mentions, or omissions), which in turn facilitates reuse and helps increase recognition, in terms of both attribution of research effort and other contributions such as the use of facilities.
• Assessable: PIDs enable reliable measurement and prediction of impact, facilitating a more strategic approach to investment, driving maximum benefit, and ensuring that valuable resources are sustained.
• Interconnected and interoperable: providing an open network of specifically identified entities supports collaboration across facilities, disciplines, institutions, and countries. This makes it easier to automate the flow of information between systems, which improves speed and accuracy in reporting and the sharing of research outputs, and can lead to discovery and innovation by exposing the links between those research outputs for anyone to use.

### An Example of PIDs in Practice

With PIDs in place, researchers can log in to a new system using credentials they already use in other research tools, for example through ORCID. Researchers can also use their ORCID ID to distinguish themselves from other researchers with the same or similar names, as well as associating multiple variations of their own name with their ID. They can pull existing publication information from databases, rather than having to re-enter it one output at a time. Their publications can be uniquely identified via a DOI, which will update if the content moves to a new URL over time; the funders of their research are clearly identified by funder and grant IDs; and their institution by their Research Organization Registry (ROR) ID, which can be pulled in automatically rather than manually entered. The inclusion of identifiers for the data that the publication references or builds on helps increase the transparency and reproducibility of the work. Combined, all of this reduces friction and increases the completeness and accuracy of records.

The presence of PIDs in the metadata associated with these records then lends itself to maximum reuse via the open application program interfaces (APIs) of PID service providers. For example, a search on a funder name linked to an identifier would return all records associated with that funder, rather than the funder having to search for a range of variations on their name over time. A search using a researcher's ORCID ID shows all their research outputs and who funded them, and results are much more accurate than searching using their name, which is unlikely to be unique.

### Supporting the Effective Use of PIDs and Metadata

It is important to note, however, that key elements need to be in place to support the implementation of PIDs and the metadata associated with them, otherwise many of the benefits described may not be realized.^14^ There are practical and technical steps involved in implementing PIDs which, if done incorrectly, can limit their effectiveness. Examples include ORCID IDs not being authenticated or verified by the researcher they relate to, DOIs being displayed but not registered with Crossref or DataCite, or poor-quality metadata being associated with any PID. A combination of factors may also mean that the community loses trust in the identifiers and becomes reluctant to spend time and effort using them and adopting them in their workflows, tools, and systems.

There remains a need for community-governed, sustainable PID providers that provide services and tools to support the entities they work with.^15^ They should be aware of issues such as these, help work to resolve them when they arise, and take steps to guard against them in future.

## The PID Graph

### PIDs and Scientific Graphs

Part of the value of PIDs springs from the attributes that are encapsulated in their very name: identification and persistence. Many of the benefits outlined above arise from the simple fact that a PID unambiguously identifies (and resolves to) an entity of interest, and that this identification is persistent over time. Equally important is that PIDs can be resolved via a URI, which follows the first principle of the Den Haag Manifesto from 2011 that tries to better align the PID and Linked Data communities.^16^

However, it is possible to go further and realize additional benefits of PIDs by connecting them together. The idea that there is something valuable in a connected graph of entities in the research domain is nothing new, of course: ever since the idea of a citation index was conceived, it has been recognized that capturing and using connections between entities leads to new insights. There are many motivations for wishing to have these insights from the perspectives of a variety of stakeholders, but some common themes emerge:

• Discoverability of all research outputs within the research ecosystem
• Reuse across versions and parts, typically of software and datasets (for example, linking these versions to pre-prints and peer-reviewed publications)
• Reuse of aggregated research outputs, providing a summary view of the reuse of all outputs of a particular individual, institution, repository, or funder
• Creating research objects, bringing together all entities linked in some way through a particular publication or other research “endpoint”
• Verification of claims made in the scientific literature by tracing the graph all the way back to its origin

The general term Scientific Knowledge Graph has been coined for such large-scale graphs, going beyond citations of published papers to encompass datasets, researchers, funding grants, and so on—precisely the kinds of entities that have PIDs. Examples are the Research Graph,^17^ the Open Research Knowledge Graph,^18^ and the OpenAIRE Research Graph,^19^ all of which describe the graph of connected scholarly resources and knowledge using a number of different approaches.

There is, however, a fundamental distinction between these graph systems and the PID Graph that has been developed within the FREYA project. In general, Scientific Knowledge Graphs take as their starting point the idea that the entities they represent are fundamental and that the construction of the graph must use whatever methods are available and practicable to discover and represent the connections. Naturally, since many of the entities have PIDs, such methods include taking advantage of these identifiers and their metadata for disambiguation and clarity of reference. In constructing such graphs, there is an emphasis on the sources of information that are indexed or harvested, and special techniques for validation and enrichment of the graph may be employed. By contrast, the PID Graph inverts this relationship and takes PIDs themselves as the basic entities that are linked together; whatever they refer to is left implicit. This approach requires that the PID metadata are sufficiently rich to represent the relationships of interest and that the PIDs are of high enough quality. The advantage is that it becomes much easier to create graphs and to implement and scale rather than working with concepts and knowledge extraction, and to trust the connections in the graph.

In this scenario, the PID metadata must not only describe entities but also the connections to other PIDs, e.g., publication to dataset (the data underlying the findings in the publication) or dataset to researchers (the creators of the dataset). Although linking of PIDs via metadata has been possible for a long time, until now this has not been done in a standardized way, so there were many gaps and the available PID connections were not made. Ideally, a PID Graph requires standard ways for exposing and discovering these connections as well as infrastructure that makes it possible to contribute and/or consume connections—which is what has been implemented as part of the FREYA project.^20^

### Implementing the PID Graph

To make the PID Graph a reality, two elements are required. First, backend services that collect PID connections in a standardized way are needed, focusing on the two PIDs that are connected and the provenance information for that connection (who, when, and so forth). This is essentially building the elements of the graph itself, ready for constructing particular subgraphs for particular purposes. Provenance information is important because of the need for assurance that the graph is well-founded on authoritative connections that can, if necessary, be validated—obviously, a graph whose origins were unclear and could not be traced could not be relied on for serious analysis and decision making. The Crossref/DataCite Event Data service,^21^ launched in 2015, is one such backend service, collecting connections between DOIs across DOI registration agencies, connections to researchers (ORCID) and funding, and connections to other PIDs and relevant URLs (e.g., mentions in Wikipedia or Twitter). The Scholix standard, which is an outcome of the RDA/WDS working group,^22^ provided the standard for exchanging information about the links between articles and datasets.

Second, query interfaces that combine this information with PID metadata must be provided. A technology that is highly suitable and has been adopted by FREYA is GraphQL. GraphQL is an open-source data query and manipulation language for APIs, and a runtime for fulfilling queries with existing data (https://graphql.org). This widely adopted query language provides a standardized interface that can be federated, making it easier to build client applications for the PID Graph. Applications built on top of the PID Graph allow users to explore the rich connections between PIDs and to address specific use cases.

**Figure 1:**
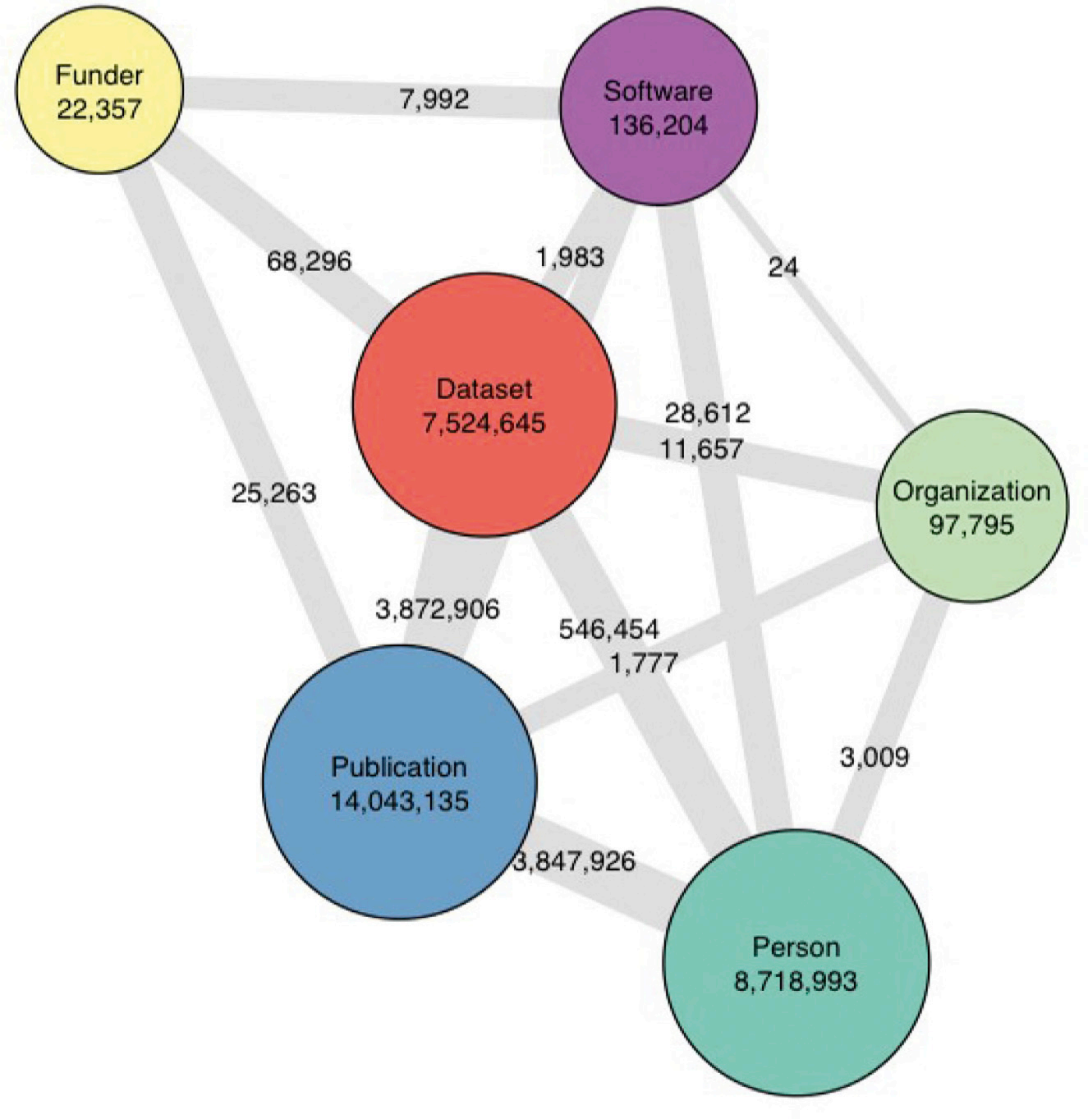
Connections between Entities in the PID Graph, August 2020

The PID Graph itself currently includes all DataCite's DOIs, 9 million Crossref DOIs, all ORCID IDs, and all ROR, Crossref Funder ID, and Registry of Research Data Repositories (re3data) records and continues to expand. Figure 1 shows the scale of the connections between these entities in August 2020. The FREYA project has produced a report summarizing the approach and implementation of the PID Graph.^23^

To illustrate the potential of the PID Graph, a number of Jupyter Notebooks have been developed to implement a range of user stories. The notebooks show how the PID Graph can be queried through the GraphQL API and can be used as a method to incentivize communities to interact with the PID Graph. The notebooks can be viewed at https://pidnotebooks.org/ and cover cases such as citations of publications based on datasets from a particular repository, reuse of research outputs from a particular institution, and types of license on outputs from work supported by a particular funder.

As a further development, an interface on top of the PID Graph was launched: the DataCite Commons. DataCite Commons provides a discovery service that enables searches while giving users a comprehensive overview of connections between entities in the research landscape, built on the PID Graph.^24^ This makes the information contained in the PID Graph available to a wide range of stakeholders in the research community.

## Recommendations: The Sum Is More Than Its Parts

Through the PID Graph, basic infrastructure is in place to answer many questions about connections within the research world. For example, funders can obtain information about all the research outputs from the research they have funded, researchers can find new datasets and software, and institutions can acquire an overview of whether their outputs are reused.

However, these questions will only have meaningful answers if sufficient information is present within the PID Graph. The following three recommendations demonstrate how different stakeholders in the community, including infrastructure providers, research institutions, repositories, publishers, funders, and researchers, can contribute meaningfully to enhancement of the Graph.

### 1. Use PIDs for All Entities

As outlined in PID Services, PIDs are available for many entities in the research process. All stakeholders can play an active role when it comes to obtaining PIDs for research entities in order to support a functioning FAIR research environment.^2^ Where there are gaps, there are often projects and working groups actively working to fill them, and it is important to remain open to the introduction of new PIDs for other types of resources.

• Researchers should use a researcher ID in their workflows, such as an ORCID ID.
• Institutions should verify and use their institution ID, e.g., a ROR ID.
• Funders should use a funder IDs and register DOIs and metadata for grants.
• Repositories and publishers should assign PIDs to outputs, such as DOIs.

### 2. Track and Record Connections between PIDs

An important part of the use of PIDs is the accompanying metadata, which provide an opportunity to document information about related identifiers. Data citation is a good example of this. It is important that an author cites a dataset and includes a reference in the reference list of an article. Other examples include researchers updating their ORCID profile and institutions including ROR IDs in the metadata of outputs in their institutional repository.

• Infrastructure providers should provide a relevant metadata schema.
• Repositories and publishers should ask for information about connections between entities.
• Researchers and institutions should include and update the information about relations wherever possible.

### 3. Make Connections Openly Available

Once relations between entities have been established through the connections between PIDs, it is important that this information becomes openly available. Open infrastructure providers play a role here by aggregating information from their members and ensuring that it is shared with the community. This means that organizations can work with their relevant infrastructure hub, such as DataCite, Crossref, or OpenAIRE. In the example above, publishers should not only be including information about links between articles and datasets in the article metadata but should also be passing this on to Crossref, from where it can feed into the PID Graph so that it can be accessed by the whole community. Although the infrastructures are in place, there are still barriers to adoption.^25^

• Infrastructure providers should aggregate the information and make it openly available.
• Publishers and repositories should ensure the information is included in the metadata they share.

Following these recommendations will help expand the PID Graph, thereby optimizing the use and value of PIDs within the research ecosystem and opening the door for valuable novel-use cases and practices. The PID Graph demonstrates that we can gain more from PIDs when we look at their connections; the sum is more than its parts.
